# Visual experience modulates the sensitivity to the distributional history of words in natural language

**DOI:** 10.3758/s13423-024-02557-6

**Published:** 2024-08-22

**Authors:** Giorgia Anceresi, Daniele Gatti, Tomaso Vecchi, Marco Marelli, Luca Rinaldi

**Affiliations:** 1 Department of Brain and Behavioral Sciences, University of Pavia, Piazza Botta 6, 27100 Pavia, Italy; 2 Cognitive Psychology Unit, IRCCS Mondino Foundation, Pavia, Italy; 3 Department of Psychology, University of Milano-Bicocca, Milano, Italy; 4 NeuroMI, Milan Center for Neuroscience, Milano, Italy

**Keywords:** Blindness, Visual experience, Linguistic experience, Distributional semantics, Semantic memory

## Abstract

Different experiential traces (i.e., linguistic, motor, and perceptual) are likely contributing to the organization of human semantic knowledge. Here, we aimed to address this issue by investigating whether visual experience may affect the sensitivity to distributional priors from natural language. We conducted an independent reanalysis of data from Bottini et al., in which early blind and sighted participants performed an auditory lexical decision task. Since previous research has shown that semantic neighborhood density—the mean distance between a target word and its closest semantic neighbors—can influence performance in lexical decision tasks, we investigated whether vision may alter the reliance on this semantic index. We demonstrate that early blind participants are more sensitive to semantic neighborhood density than sighted participants, as indicated by the significantly faster response times for words with higher levels of semantic neighborhood density shown by the blind group. These findings suggest that an early lack of visual experience may lead to enhanced sensitivity to the distributional history of words in natural language, deepening in turn our understanding of the strict interplay between linguistic and perceptual experience in the organization of conceptual knowledge.

## Introduction

The development and organization of semantic knowledge in humans is a complex process that involves a strict interplay between perceptual and linguistic experiences (Andrews et al., 2009; Jones et al., 2015a; Louwerse, 2018). However, the exact nature of this interaction is a matter of a fervent debate (Davis & Yee, 2021; Lupyan & Lewis, 2019). According to traditional embodied theories of cognition, semantic representations are grounded and intrinsically rooted in the body’s sensorimotor interactions with the world (Barsalou, 2008; Wilson, 2002; Feldman & Narayanan, 2004). On its most radical formulation, this view posits a complete dependence of conceptual understanding on reenacted sensorimotor circuits through simulation, while ascribing only a marginal role to linguistic experience (Gallese & Lakoff, 2005). On the other hand, amodal (or symbolic) theories posit that semantic representations are achieved with the transduction of sensorimotor information into a qualitatively different format, which is purely symbolic in nature (Pylyshyn, 1985). In this perspective, semantic information can be fully captured by a language-like representational system, which is conceived as abstract and symbolic, without an inherent relationship between the form of the representation and its referent (Fodor, 1975; Meteyard et al., 2012). While the amodal and embodied accounts have been traditionally contrasted, recent views acknowledge that both sensorimortor and linguistic experiences would be crucial in the development of semantic knowledge. However, the relative contribution of each type of experiential trace is still debated (Andrews et al., 2009; Davis & Yee, 2021; Günther et al., 2023; Lupyan & Lewis, 2019). This is especially due to the fact that humans’ reliance and sensitivity to perceptual or linguistic experience may flexibly vary depending on situational and contextual demands (Kemmerer, 2015; Wingfield & Connell, 2022; Yee & Thompson-Schill, 2016).

One way to tackle this issue is by investigating how individuals who lack some perceptual input develop (and make use of) semantic knowledge. In this regard, thereby missing visual input, congenitally blind people have been shown to possess a surprising amount of knowledge about visual perceptual qualities (Bedny et al., 2019; Landau & Gleitman, 1985; Petilli & Marelli, 2024). For example, it has been suggested that from an early age, blind children can meaningfully comprehend and produce color adjectives and visual perception verbs in proper ways (Landau & Gleitman, 1985). Analogously, blind and sighted adult individuals produce comparable semantic similarity judgments of visual verbs (Bedny et al., 2019). This means that blind individuals can compensate for the lack of visual input by likely relying on the linguistic environment to grasp the meaning of concepts primarily pertaining to the visual domain. In line with this, J. S. Kim et al. (2019) showed that blind participants demonstrate extensive knowledge of the visual appearance of animals as well as a remarkable agreement with sighted participants in their judgments of animals’ size, shape, and skin texture. However, consistent with previous research investigating color understanding in blind individuals (Saysani et al., 2018; Shepard & Cooper, 1992), the lowest correspondence between the sighted and blind groups was found for animal color (Kim et al., 2019). Assuming that color properties are highly verbalizable information, the authors hence concluded that blind people may not use verbal descriptions (e.g., “crows are black”) as a primary source of information, prioritizing inferential reasoning instead (e.g., “if birds have feathers, then crows do as well”; Kim et al., 2019). However, another possibility for explaining these results is that some visual information may be less available in natural language (i.e., prototypical colors about entities may not be enough verbalized in natural language).

In this regard, distributional semantic models (DSMs) represent a powerful tool to quantify the role of linguistic experience in shaping semantic knowledge. Indeed, DSMs capture word meaning from their distributional history across large written corpora documenting natural language usage (Lenci & Littell, 2008). In addition, since their architecture is based on associative learning mechanisms and lacks a direct inferential algorithm, any representation reproduced from these models is language-derived and noninferential by definition. On this matter, it is crucial to acknowledge that being devoid of explicit inferential machinery does not preclude DSMs from containing reliable inferential knowledge (see Peterson et al., 2020). However, unlike explicit inferential mechanism, DSMs do not directly infer meaning from logical rules or deductions. Instead, inferential knowledge captured by these models is derived indirectly from statistical patterns available in the language data. The theoretical foundation of this approach indeed lies in the distributional hypothesis, according to which similar words will tend to appear in similar linguistic contexts (Harris, 1954). DSMs operationalize this assumption by providing a mathematical encoding of the distributional history of words. That is, in DSMs, words are represented as high-dimensional numerical vectors populating a common multidimensional (semantic) space, and the distance (indexed by the cosine of the angle) between such vectors is conceived as a proxy for their semantic similarity. That is, the closer the vectors in the semantic space, the higher the semantic similarity of the corresponding words (Lenci, 2018). Importantly, since DSMs build on cognitively plausible associative learning models (Günther et al., 2019; Mandera et al., 2017) they can be conceived as a computationally implemented framework of the human semantic memory. Supporting this, DSMs have indeed been found to predict human performance in a variety of tasks among which semantic priming (Günther et al., 2016; Jones et al., 2015a, 2015b; Lapesa & Evert, 2013; Lund & Burgess, 1996) and false memories paradigms (Gatti et al., 2022); with data extracted from these models strongly correlating with human semantic similarity ratings (Baroni et al., 2014; Landauer & Dumais, 1997).

Crucially, by making use of DSMs, Lewis and colleagues (2019) showed that such language-derived semantic representations significantly predicted both blind and sighted participants’ judgments of animal characteristics reported by Kim et al. (2019). Yet, animal color judgments were captured by DSMs to a lower extent as compared with shape judgments (likely because in text corpora, few authors will write rather obvious statements such as “a strawberry is red”; see Bruni et al., 2014), and only for the blind group. Thus, even if one acknowledges that DSMs’ representations of attributive properties (e.g., color) are less accurate than taxonomic properties (e.g., *being red vs. being a fruit*; Rubinstein et al., 2015; see also Ostarek et al., 2019), these results still challenge the view that blind people do not (largely) rely on (distributional) linguistic experience when representing visual knowledge. Rather, the fact that DSMs capture animal color judgments only for blind participants (although with relatively a small effect) suggests that being devoid of visual input may result in an enhanced reliance on prior linguistic knowledge (Giraud et al., 2023).

Whereas previous studies have mostly relied on explicit behavioral measures such as ratings or sorting tasks (e.g., Bedny et al., 2019; Kim et al., 2019; Shepard & Cooper, 1992) one way to further explore whether blind individuals rely more on linguistic experience (and, specifically, on distributional language learning) is by assessing participants’ chronometric performance in computerized tasks. To do so, we conducted an independent reanalysis of the data reported by Bottini and colleagues (2022), in which early blind and sighted participants performed an auditory lexical decision task. In this task, indeed, it has been shown that participants’ performance is typically modulated by a semantic index quantifying the average semantic similarity among each word and its *k* most similar words (Buchanan, et al., 2001; Hendrix & Sun, 2021; Yap et al., 2015). This measure builds on the distributional history of words in linguistic contexts and captures the density of the neighborhood (in terms of words) as represented in the semantic space (Hendrix & Sun, 2021; Yap et al., 2015). Indeed, previous research suggested that word recognition in lexical decision tasks is faster when lexical stimuli are located in dense semantic neighborhoods (Brysbaert et al., 2018; Buchanan et al., 2001; Yap et al., 2015). However, evidence of an enhanced influence of such a linguistic effect in the context of blindness is missing. Here, we aimed to address this possibility by exploiting DSMs. Specifically, we investigated whether the missing visual experience in the blind may influence their reliance on linguistic experiential priors such as semantic neighborhood density as indexed through distributional models (e.g., Hendrix & Sun, 2021) when performing a lexical decision task. The use of distributional models is particularly convenient in this case as it allows quantifying the role of (a proxy for) the linguistic experience.

We hypothesized that, if being devoid of visual input results in an increased reliance on linguistic experience, blind participants should show an enhanced sensitivity to semantic neighborhood density than sighed participants. This would be reflected in faster response times for words with denser semantic neighbors in the blind as compared to the sighted group.^1^

## Methods

### Participants

We conducted an independent reanalysis of the data reported by Bottini and colleagues (2022; available at https://osf.io/5gtjw), in which early blind and sighted participants performed an auditory lexical decision task. Our independent reanalysis is available online (https://osf.io/a9wu5/). The study involved 42 participants (21 early blind individuals [EB]; 21 sighted controls [SC]). EBs and SCs were native Italian speakers, matched pairwise for gender, age, and years of formal education. All the EB participants lost their sight completely at birth or before the age of three and reported no visual memories.

### Materials and procedure

Stimuli in the study by Bottini and colleagues (2022) included 120 adjectives (40 abstract, 40 concrete multimodal, and 40 concrete unimodal visual words) selected from an Italian database of modality exclusivity norms (Morucci et al., 2019). For each word, a corresponding pseudoword was created using the software Wuggy (Keuleers & Brysbaert, 2010). Both words and pseudowords stimuli were then synthesized with an artificial female voice (TalkToMe software), as the stimuli were presented in the auditory modality. The lexical decision task required participants to decide as fast and as accurately as possible whether the stimulus was a word or not by pressing one of two response keys. The full set of stimuli was played two times for each participant, for a total of 480 trials presented in random order.

### Distributional semantic model

The DSM used was *fastText* (Joulin et al., 2016), and word vectors were retrieved from the Italian pretrained vectors (Grave et al., 2018). The model was trained on Common Crawl and Italian Wikipedia (around 11 billion words) using the Continuous Bag of Words (CBoW) method (Mikolov et al., 2013) with 300 dimensions, character *n*-grams of a length of 5, and a window of size 5. When using CBoW, the obtained vector dimensions capture the extent to which a target word is predicted by the contexts in which it appears. With respect to traditional distributional models, whose ability to generate high-quality distributed semantic representations is limited to words that are sufficiently frequent in the input data, *fastText* is based on the idea (originally proposed by Schutze, 1993; and realized by Bojanowski et al., 2017) to take into account sub-word information by computing word vectors as the sum of the semantic vectors for the n-grams associated with each word.

Using *fastText* we thus obtained vector representations for all 120 word stimuli, together with the vector representations for the 20,000 most frequent words in SUBTLEX-it (Crepaldi et al., 2015), to be used for the computation of the semantic neighborhood density index.

### Data analysis

All the analyses were performed with R-Studio (RStudio Team, 2015). For each word stimulus, we computed an index of semantic neighborhood density (hence, SNeigh), as estimated via DSMs. Following the methodology adopted by Hendrix and Sun (2021), we first estimated the cosine of the angle between the vectors representing the meanings of each of the 120 words included in the study and those representing the meanings of the 20,000 most frequent words in the SUBTLEX-it (Crepaldi et al., 2015). The cosine indeed is typically taken as a proxy for semantic similarity (Günther et al., 2019): The higher the cosine value, the more semantically related the words are expected to be.

**Fig. 1:**
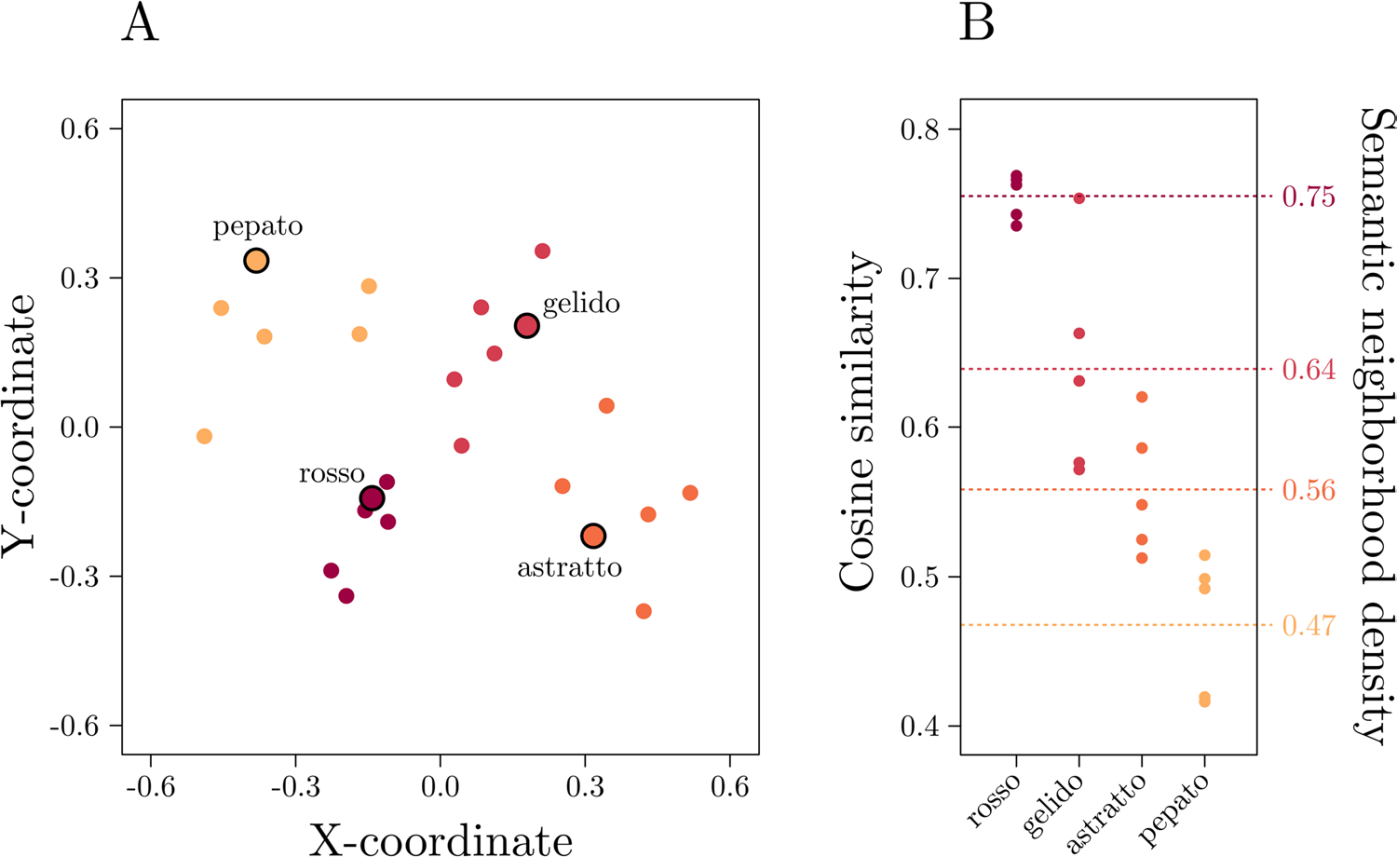
**A** Scatterplot representing four example words (selected among the list of stimuli used) and their five closest neighbors as resulting from an isoMDS procedure (i.e., a procedure that, given a matrix of distances among items, provides their coordinates; Venables & Ripley, 2002). **B** Plot representing the cosine similarities among the four example words and their five closest neighbors and the density of their semantic neighborhood (the mean of the cosine similarities; right labels). Warmer colors indicate that, for a given example word, the neighborhood is denser. (Color figure online)

Then, for each of the 120 words, SNeigh was operationalized as the mean cosine similarity between each word and its *k* closest neighbors (with *k* = 5; see Hendrix & Sun, 2021; excluded the word itself). Hence, a higher SNeigh value indicates a denser semantic neighborhood (see Fig. 1 for a graphical representation). Additionally, for each stimulus, we included log-transformed stimulus duration as a predictor, which was retrieved from Bottini and colleagues’ (2022) dataset.

Using the *lme4* R package (Bates et al., 2015), we estimated a linear mixed model. Log-transformed correct response times (RTs) were included as dependent variable, SNeigh, and log-transformed stimulus duration were included as continuous predictors, Type (abstract, multimodal, visual) and Group (EB, SC) as categorial predictor, along with the interactions of Group with SNeigh and Type.^1^ Participants and stimuli were set as random intercepts, and SNeigh over participants was included as random slope. Specifically, in the *lme4* syntax the model estimated was:

$$log\left(RTs\right) \sim Group\times \left(SNeigh +Type\right) +\text{log}\left(duration\right)+ (SNeigh|participant) + (1|stimulus)$$

In the Results section, we report the results of this estimated model. As a last sanity check, after fitting the model, to exclude the impact of overly influential outliers, we also checked whether the observed effects were significant also when removing data points based on a threshold of 2.5 *SD* standardized residual errors (model criticism; see Baayen et al., 2008).

## Results

Consistent with Bottini and colleagues’ (2022) data-cleaning procedures, we initially examined the overall accuracy at both the participant and item levels to identify outliers. At the participant level, we did not detect outliers in the SC group. However, in line with Bottini and colleagues, one participant was excluded from the EB group due to low accuracy, with an error rate that was more than 2.5 *SD* higher than the other EB participants. At the item level, three words were excluded due to an accuracy rate of 2.5 *SD* lower than the average rate in both groups. Then, inaccurate trials (1.75%) as well as trials in which participants’ RTs were faster than 300 ms (.02%) were removed from the analysis. Finally, trials in which RTs were ±3 *SD* from the mean RTs of each participant (1.42%) were excluded from the analysis.^2^

Results showed a main effect of Group, *F*(1, 39) = 9.40, *p* = .004, duration, *F*(1, 111) = 96.56, *p* < .001, Type, *F*(2, 111) = 4.29, *p* = .016, and SNeigh, *F*(1, 116) = 12.10, *p* < .001. The main effect of duration indicated that shorter stimulus durations were associated with faster RTs, *b* = .25, *SE* = .03. Regarding the main effect of Type, post hoc pairwise comparisons revealed that the abstract words elicited slower response times compared with both multimodal, *z* = 2.58, *SE =* .01, *p* = .01, and visual words, *z* = 2.54, *SE* = .01, *p* = .01, while no significant difference emerged between visual and multimodal words, *z* = .05, *SE* = .01, *p* = .96. These results are in line with the literature on the concreteness advantage effect (Allen & Hulme, 2006; Kroll & Merves, 1986; Schwanenflugel & Stowe, 1989) and with the results of Bottini and colleagues (2022). Consistently with the results reported by Bottini and colleagues, the Group × Type interaction was not significant, *F*(2, 9096) = 2.24, *p* = .11.

**Fig. 2.**
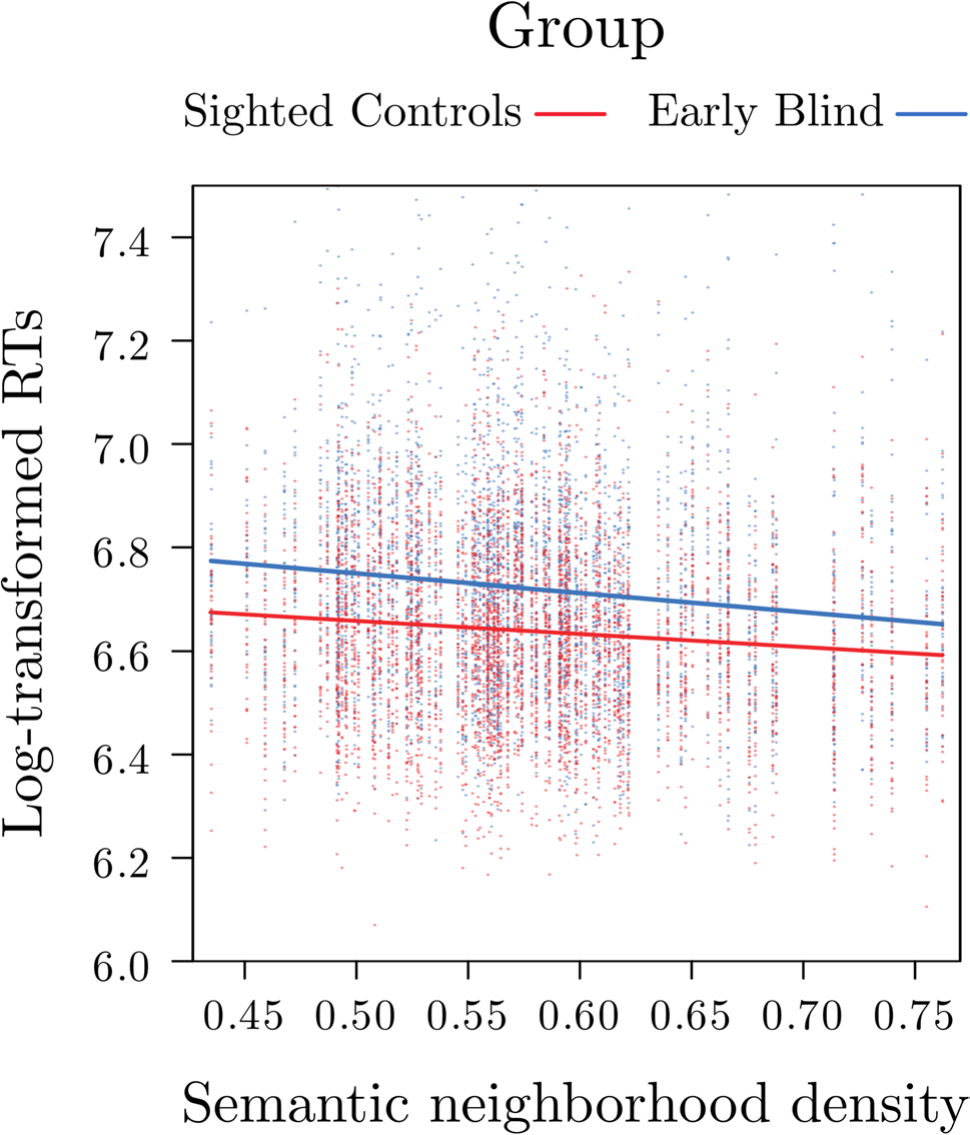
Plot illustrating the interaction between group and semantic neighborhood density on reaction times. In particular, words with higher (i.e., denser) semantic neighborhoods were recognized faster than words with lower semantic neighborhoods, with this effect being significantly stronger for the early blind participants as compared to sighted participants. (Color figure online)

Crucially, the Group × SNeigh interaction was significant, *F*(1, 39) = 4.17, *p* = .047, indicating that the effect of SNeigh on RTs differed between the two groups (see Fig. 2). It is worth noting that the inclusion of the random slope of SNeigh over participants accounts for individuals’ variation around the mean effect of SNeigh. This interaction effect indicates that, although for both groups the higher the SNeigh (and thus the denser the neighborhood) the faster the RTs, this effect is stronger for the EB group, *b* = −.37, *SE* = .09, *z* = −3.93, *p* < .001, as compared with the SC group, *b* = −.25, *SE* = .09, *z* = −2.69, *p* = .007. These results hold against model criticism based on a threshold of 2.5 *SD* standardized residual errors, with a significant interaction Group by SNeigh also after applying this procedure, *F*(1, 37)* =* 9,71*, p = .*025 (Baayen et al., 2008), see Table 1.

**Table 1:** Regression table including the results of the LMM on RTs including fixed and random effects variances (as recommended in Meteyard & Davies, 2020)

*FIXED EFFECT*	*Sum sq*	*Mean sq*	*F value*	*NumDF, DenDF*	*p value*
Group	0.21	0.21	9.40	1, 39	**.004**
SNeigh	0.27	0.27	12.10	1, 116	**<.001**
Type	0.19	0.09	4.29	2, 111	**.016**
Log(duration)	2.15	2.15	96.56	1, 111	**<.001**
Group : SNeigh	0.09	0.09	4.17	1, 39	**.047**
Group : Type	0.10	0.05	2.24	2, 9096	.107
***RANDOM EFFECT***	***Variance***		***SD***		***Correlation***
Stimulus (intercept)	0.003		0.06		
Participant (intercept)	0.02		0.13		
SNeigh (slope)	0.01		0.11		-0.50
***MODEL FIT***	***Marginal***		***Conditional***		
*R*^2^	0.14		0.50		

## Discussion

In the present study, we re-analyzed data from Bottini and colleagues (2022) to investigate whether vision (and the absence thereof) can shape humans’ reliance on linguistic experience in the organization of semantic knowledge. In particular, we exploited DSMs to assess whether blind and sighted participants’ performance in a lexical decision task differently relied on semantic neighborhood density—a measure tackling the semantic density of a word with respect to neighbors’ words in its semantic space. In line with our hypothesis, results showed that blind individuals are more sensitive to this semantic index, as reflected by faster response times for words with denser neighborhoods observed in the blind as compared with the sighted group.

These findings contribute to ongoing debates about how perceptual and linguistic sources of knowledge interact in the development of semantic representations (Andrews et al., 2009; Davis & Yee, 2021; Lupyan & Lewis, 2019). Specifically, on the one hand, embodied perspectives postulated a close connection between conceptual representations and their sensorimotor characteristics, while on the other hand, amodal accounts conceived knowledge as fully detached from the perceptual and motor components active during the encoding of information (Davis & Yee, 2021; Meteyard et al., 2012). However, both theoretical positions have faced significant criticism. A major argument advanced against (radical) embodied views is that the knowledge gained through perceptual experience is limited to concrete concepts with tangible physical referents (cf. Borghi et al., 2017). Additionally, this perspective would predict substantial differences in the representations of world knowledge between individuals with diverse perceptual experiences, such as sighted and blind individuals. Contrary to this prediction, several studies demonstrated that blind and sighted individuals show remarkable similarities in conceptual representations both at the behavioral (Bedny et al., 2019; Landau & Gleitman, 1985; Zimler & Keenan, 1983) and neural level (Mahon et al., 2009; Noppeney et al., 2003). On the other side, the major theoretical concern generated by amodal theories refers to the challenge of symbol grounding—that is, the possibility to establish a meaningful connection between abstract symbols (i.e., words) and the concepts they refer to. In other words, for abstract symbols to accurately represent meanings, they would have to ultimately relate back to their underlying conceptual (concrete) referents. Although recent perspectives acknowledge that these theories should not be treated as mutually exclusive, and that perceptual and linguistic knowledge reciprocally reinforce each other (Binder & Desai, 2011; Günther et al., 2023), the precise nature of their interactions and the circumstances under which individuals may flexibly rely more on each type of experience to varying degrees remain a topic of ongoing debate (Andrews et al., 2009; Davis & Yee, 2021; Louwerse, 2018). Importantly, interactions between embodied and amodal processes of word meaning have been discussed also at the neural level (Bi, 2021; Carota, et al., 2012; Vignali et al., 2023; Xu et al., 2017). For example, recent evidence exploring the temporal dynamics of semantic representations suggests that the concreteness of words would be first processed with a symbolic code, followed by a later sensorimotor code (Vignali et al., 2023).

Within this context, blindness provides valuable insights to explore the interplay between perceptual and linguistic experiences in shaping knowledge. Remarkably, it has been shown that, even in the absence of direct visual sensory access, congenitally blind individuals are able to retrieve a surprising amount of information about visual perceptual qualities (Bedny et al., 2019; Landau & Gleitman, 1985; Zimler & Keenan, 1983). This raises the question of how this knowledge develops in blind individuals. To answer this question, Kim and colleagues (2019) suggested that blind individuals may acquire visual knowledge primarily through inference. However, the results presented here arguably support an alternative interpretation. While it is not possible to deny the significance of inferential reasoning in generating knowledge, the present findings demonstrate that the (noninferential) distributional history of words in natural language could serve as a primary source of information too. Interestingly, previous studies have shown that DSMs, although being built on a noninferential architecture, are able to produce reliable inferences about the world and the entities populating it (e.g., Berlin : Germany = Rome : x; see Peterson et al., 2020). As such, a distributional model of semantic knowledge may serve as the basis for solving inferential tasks. However, it should be acknowledged that inferential knowledge produced by DSMs is derived indirectly from distributional patterns in the data rather than through explicit inference mechanisms (i.e., logical rules or deductions).

Notably, different cognitive mechanisms and theoretical models of inferential reasoning have been proposed (Hayes et al., 2010, 2018). A robust finding in the literature highlights the key role of perceived similarity among concepts for the transmission of properties between them (Hayes et al., 2010). Moreover, research suggests that inferential reasoning based on similarity emerges early in life, potentially representing a foundational principle guiding the development of inferential processes (Keates & Graham, 2008; López et al., 1992). In light of this, an intriguing possibility is that the ability of DSMs to capture the semantic similarity between concepts—by tracking into their distributional patterns on natural language—might itself act as an indirect (and implicit) generator of inference. For instance, the similar distributional history shared between “crow” and “bird” and between “bird” and “sparrow” could serve as a foundation to infer that “crow” and “sparrow” are likely to share similar properties. In this regard, inferential knowledge may emerge across different concepts and model architectures. Hence, it is plausible that this capability reflects an inherent process related to distributive data itself (Landauer & Dumais, 1997). Overall, the findings reported here may therefore suggest that these two processes are not mutually exclusive, but rather that they interact and compensate each other depending on the context, on the task and on the (sensorimotor) resources available.

It is also crucial to note that, even without awareness, humans possess a remarkable ability to detect statistical regularities from the environment, and to develop structured knowledge from the continuous flow of information they are exposed to (Palmer et al., 2018; Sherman et al., 2020; Smith & Yu, 2008). This ability spans across a variety of domains, making humans potentially able to rely on multiple experiential traces from different modalities (i.e., perceptual and linguistic). Indeed, it has been suggested that similar principles guiding the acquisition of knowledge from the exposure to language statistics apply also to perceptual experiences (Andrews et al., 2009; Davis & Yee, 2021). This perspective is consistent with a comprehensive view of knowledge development and organization driven by an experience-dependent plasticity. When a sensory modality is absent, other experiential traces may become more relevant in order to compensate for the missing one, as observed, for instance, in the heightened sensitivity showed by blind individual to sounds detection (Ashmead et al., 1998; Niemeyer & Starlinger, 1981; Nilsson & Shenkman 2016). Crucially, since language is essentially used to share with others information about the perceptual world we live in, a substantial amount of perceptual information is actually encoded in language, so that the perceptual and linguistic environment give rise to interconnected and mutually reinforcing sources of knowledge (Günther et al., 2019; Louwerse, 2011). In this regard, the value of DSMs relies in their ability to quantify the specific role played by distributional patterns of the linguistic environment in conveying word knowledge, which we found to become more relevant when other perceptual inputs, as in the case of blindness, are missing. Indeed, DSMs are built on cognitively plausible associative learning models (Günther et al., 2019; Mandera et al., 2017), and provide a mathematical encoding of the distributional history of words, which are ultimately represented by high-dimensional numerical vectors in a common multidimensional (semantic) space. The semantic neighborhood density index captures the semantic density of words, building on their distributional history (Buchanan et al., 2001; Hendrix & Sun, 2021; Yap et al., 2015). Crucially, the enhanced sensitivity to semantic neighborhood density showed by blind individuals demonstrates that distributional linguistic experience plays a direct role in organizing (their) semantic knowledge, thus becoming a much more relevant source when other traces (e.g., visual input) are not available. These results align with prior research demonstrating a facilitatory effect of semantic neighborhoods on word recognition, where stimuli situated in dense semantic neighborhoods are associated with faster recognition times (Brysbaert et al., 2018; Buchanan et al., 2001; Hendrix & Sun, 2021; Yap et al., 2015). Notably, it is worth recognizing that, although here we adopted a distributional approach on the study of semantic memory, other approaches—mainly related to the extraction of vector representations as emerging from human-based perceptual norms (e.g., Lynott et al., 2020; Vergallito et al., 2020)—could have been employed in a similar way (e.g., Wingfield & Connell, 2023). However, this choice would not align with our aim to use an independent measure that is purely linguistic and data-driven. When feasible, this methodology is desirable for psychological studies as it avoids the circular process of explaining behavioral data (e.g., as for the case of reaction times in a lexical decision task) with other behavioral data modeled for specific, task-related purposes (as for the case of norms or human ratings; for a discussion see, Günther et al., 2023; Jones et al., 2015a, 2015b; Petilli & Marelli, 2024; Westbury, 2016). Indeed, studies using human ratings (or human-based measures) to predict human behavior risk to leave our understanding of the process at hand at the same level of description of the predictor(s) without actually addressing the cognitive phenomenon of interest. In other words, using task-related behavioral data to quantitatively operationalize mental representations keeps the explanation at the same phenomenological level as the object of investigation, thereby conflating the explanation with the phenomenon itself.

Moreover, from a theoretical point of view, the main rationale behind our choice to employ a purely language-based approach stems from our specific aim to assess and quantify the impact of (a proxy for) the linguistic experience. However, it is crucial to recognize that, while DSMs receive text as input and efficiently capture linguistic experience as a fundamental source of conceptual knowledge, individuals construct mental representations incorporating various modalities (Andrews et al., 2009; Meteyard et al., 2012). Therefore, in order to develop more comprehensive models of human semantic memory, future research should integrate complementary models that can also capture perception-based representations (see, e.g., Günther et al., 2020, 2023). Overall, these findings suggest a different reliance on linguistic experience in the development of semantic knowledge for blind individuals as compared with sighted participants. This study thus supports the existence of a strict interplay between perceptual and linguistic sources of knowledge and contributes to the broader theoretical discussions about the nature of the circumstances under which humans may flexibly rely more on each type of experience. Specifically, knowledge of the world may be acquired by bootstrapping from distributional data within language itself, and we demonstrate that individuals lacking visual perceptual input exhibit an enhanced reliance on linguistic experiential priors.

## Data Availability

All data, scripts, codes, and materials used in the analysis are available online (https://osf.io/a9wu5/). The study was not preregistered.

## Footnotes

1 Word frequency was dropped from the model due to multicollinearity issues. This was tested by computing the variance inflation factors (VIF) for each predictor (Allison, 1999). Word frequency showed a VIF value >2.5, thus indicative of considerable collinearity (Johnston et al., 2018), while all the other predictors showed <2.5 VIF values.

2 Note, however, that comparable results were observed also when including these trials.
